# The Feasibility of the Interferon Gamma Release Assay and Predictors of Discordance with the Tuberculin Skin Test for the Diagnosis of Latent Tuberculosis Infection in a Remote Aboriginal Community

**DOI:** 10.1371/journal.pone.0111986

**Published:** 2014-11-11

**Authors:** Gonzalo G. Alvarez, Deborah D. Van Dyk, Naomi Davies, Shawn D. Aaron, D. William Cameron, Marc Desjardins, Ranjeeta Mallick, Natan Obed, Maureen Baikie

**Affiliations:** 1 Ottawa Hospital Research Institute, University of Ottawa, Divisions of Respirology and Infectious Diseases, Departments of Medicine and Microbiology, The Ottawa Hospital, Ottawa, Ontario, Canada; 2 Government of Nunavut, Department of Health, Iqaluit, Nunavut; 3 Nunavut Tunngavik Inc, Iqaluit, Nunavut; Food and Drug Administration, United States of America

## Abstract

**Background:**

The tuberculin skin test (TST) is the standard test used to screen for latent TB infection (LTBI) in the northern Canadian territory of Nunavut. Interferon gamma release assays (IGRA) are T cell blood-based assays to diagnose LTBI. The Bacillus Calmette-Guerin (BCG) vaccine is part of the routine immunization schedule in Nunavut. The objective of this study was to test the feasibility, and predictors of discordance between the Tuberculin Skin Test (TST) and the IGRA assay in a medically under-serviced remote arctic Aboriginal population.

**Methods:**

Both the TST and QuantiFERON-TB Gold (Qiagen group) IGRA tests were offered to people in their homes as part of a public health campaign aimed at high TB risk residential areas in Iqaluit, Nunavut, Canada. Feasibility was measured by the capacity of the staff to do the test successfully as measured by the proportion of results obtained.

**Results:**

In this population of predominantly young Inuit who were mostly BCG vaccinated, the use of IGRA for the diagnosis of LTBI was feasible. IGRA testing resulted in more available test results reaching patients (95.6% vs 90.9% p = 0.02) but took longer (median 8 days (IGRA) vs 2 days (TST), p value <0.0001). 44/256 participants (17.2%) had discordant results. Multivariable regression analysis suggested that discordant results were most likely to have received multiple BCG vaccinations (RR 20.03, 95% CI, 3.94–101.82)), followed by BCG given post infancy (RR 8.13, 95% CI, 2.54–26.03)) and then to a lesser degree when BCG was given in infancy (RR 6.43, 95% CI, 1.72–24.85).

**Interpretation:**

IGRA is feasible in Iqaluit, Nunavut, a remote Arctic community. IGRA testing results in more test results available to patients compared to TST. This test could result in fewer patients requiring latent TB treatment among those previously vaccinated with BCG in a region with limited public health human resources.

## Introduction

Located in the Canadian arctic, the Territory of Nunavut has the highest incidence rate of tuberculosis (TB) in Canada [1]. Screening and treatment of latent TB infection (LTBI) is a part of the public health strategy to reduce the number of active TB cases in Nunavut [2]. Treatment of latent TB infection can significantly decrease the risk of developing active TB disease [3]. The tuberculin skin test (TST) is the standard test used to screen for LTBI.

Interferon gamma release assays (IGRA) are T cell based assays that produce interferon gamma when re-exposed to TB specific antigens in the blood ESAT-6, CFP-10 and TB7.7 (p4) proteins [1], [4]. IGRAs use specific antigens found only in TB that are not found in Bacillus Calmette-Guerin (BCG) vaccine or in most non tuberculous mycobacteria [4], [5]. Additional advantages of these assays over the TST are that they do not require the operator to be trained in skin test administration and interpretation since they are blood based assays done in a laboratory and no return visit is required to interpret the result. Potential disadvantages to the use of the IGRA, particularly in a remote area such as Nunavut, include indeterminate results, phlebotomy challenges in young children [6], cost [7], and the need for laboratory expertise to process and analyze the test [1] in a remote geographical area.

Nunavut is one of the few places in Canada where BCG is offered in infancy due to the high incidence of active TB disease. The TST is the standard of care for screening for LTBI in Nunavut. However, the TST's specificity is low and variable in BCG vaccinated populations [8]. Furthermore, a significant lack of human resources in both public health and laboratory services exists in Nunavut. We hypothesized that the IGRA, which involves a single blood draw, might provide advantages over the TST to diagnose TB infection in a medically underserved arctic Inuit population at relatively high risk for TB. The objectives of our study were to test the feasibility, of the introduction of the IGRA assay compared to the TST and to determine predictors of discordance between the TST and IGRA in a high-risk population in Iqaluit, Nunavut.

## Methods

### Setting and participants

Iqaluit, the capital of the territory of Nunavut, is located in the Canadian arctic and can only be accessed by plane during the winter months and ship or plane during the brief summer. The local hospital laboratory does not have any TB testing capacity. Samples are flown to the nearest major center (Ottawa, Canada) for testing at a private laboratory. Between January 2011 and February 2013, a TB prevention campaign (TAIMA (Stop) TB) was carried out by the investigators in Iqaluit, Nunavut. The campaign involved various community TB awareness activities followed by a door to door screening and testing campaign in residential areas of high risk for TB. A TB champion supported by a public health nurse did the door-to-door screening during working hours, Monday to Friday. The TB champions were local Inuit community members trained in TB education by nursing staff. TB messaging produced by local health care providers and community members was used to produce videos done by community members. The videos were then used as a vehicle to support the oral Inuit tradition for the sharing of information. The TB champions then presented these videos to each household participant in their language of choice (English or Inuktitut) using a portable DVD player. This format allowed messaging to be delivered in a standardized and reproducible manner. Discussion and questions surrounding TB, including the signs and symptoms of active TB, were done in Inuktitut and/or English in the home [9]. As part of the door to door campaign, persons over the age of 6 months were included in the study and offered LTBI screening in their homes if they provided written informed consent (parents provided consent for children under 16). Participants with no record of previous TST or a record of previous negative TSTs were offered both the TST and IGRA QuantiFERON^©^-TB Gold (Qiagen group). Participants were excluded from testing if they had been previously treated for either or both latent TB infection or active TB disease. Forty-five participants had a known previous positive TST but had not received treatment; we did not repeat a TST in these subjects and they were only tested with the IGRA. These subjects were considered TST positive for the analysis.

### TST testing

Participants had blood drawn for IGRA first then a TST was implanted. The TST was injected intradermally into the forearm with purified protein derivative (5 tuberculin units) and read 48 – 72 hours later as per Canadian standards [1]. Nurses made up to three separate attempts to read the TST result by going to the participant's home or phoning the participant to organize a meeting. The TST was considered positive ≥10 mm induration.

### IGRA testing

Blood was drawn for IGRA testing then shaken immediately to ensure that the entire inner surface of each tube was coated with blood. Most participants had their blood drawn in the home, however young children and others from whom blood was unable to be drawn at home were sent to the laboratory for phlebotomy. Blood was incubated within 8 hours of collection and then put in the incubator at 37C for 16–24 hours. Tubes were then centrifuged in Iqaluit and then flown to a reference laboratory in Ottawa for testing using the ELISA QuantiFERON^©^-TB Gold (Qiagen group). IGRA was considered positive if >0.35 IU/mL. If the IGRA was indeterminate, blood was redrawn and tested again. The nurses were blinded at the time of reading of the TST to the IGRA results, and the laboratory personnel carrying out the IGRA were blinded to the TST results. If either test was positive the participants were offered treatment. Nine months of isoniazid (INH) given twice weekly directly observed as per Nunavut policy was offered to all those with positive tests. Ethnicity was determined by land claim beneficiary status.

### Main measurements and data analysis

Feasibility was defined as the extent to which the IGRA could be successfully used by the staff and patients in the Canadian Arctic setting of Iqaluit, Nunavut as has been suggested in other disciplines [10]. Feasibility was measured by the capacity for the public health team and laboratory staff to do the test successfully as measured by the proportion of results obtained and the proportion of indeterminate tests as done in a previous study [6]. The time to obtain a result was measured as the time the specimen was obtained by a blood draw (IGRA) or the implantation of the TST to the time the participant was given the results. This end point was chosen so that the TST and IGRA turnaround times could be compared because the TST result is given to the patient at the time it is read. Previously positive TSTs were not included in the turnaround time calculation because the results were not obtained during this study. No gold standard is available for LTBI therefore IGRA was compared to TST. Test discordance was defined as TST positive/IGRA negative or TST negative/IGRA positive. Predictive models were generated to identify risk factors for test discordance. Discordant results were analyzed separately in the sensitivity analysis however only TST+/IGRA- were included in the final analysis as they are the most clinically relevant and the impact of doing the analysis with TST-/IGRA+ was negligible as they represent a rare event both in the study and in the literature. Discordant groups were compared to the TST negative/IGRA negative group in the development of the models as has been done in previous studies [11], [12]. Univariable predictors were assessed and multiple logistic regression models were constructed using age, gender, ethnicity, repeated TSTs and BCG vaccination status as independent variables and test discordance as the dependent variable. There were 4 categories of BCG status: no BCG, BCG given at infancy, BCG given post infancy and BCG given multiple times. Accordingly a categorical variable was created where each participant falls into only one category and was added as a covariate in the model. Sensitivity analyses were done with discordant pairs compared to the both concordant negatives and positives. BCG vaccination was recorded as positive only if the subject's health records indicated that the vaccine had been previously given since 20–50% of BCG vaccine recipients will fail to develop a scar [13], [14]. Ethics approval was obtained from the Ottawa Hospital Research Ethics Board and the Public Health Agency of Canada Research Ethics Board.

## Results

**Figure 1:**
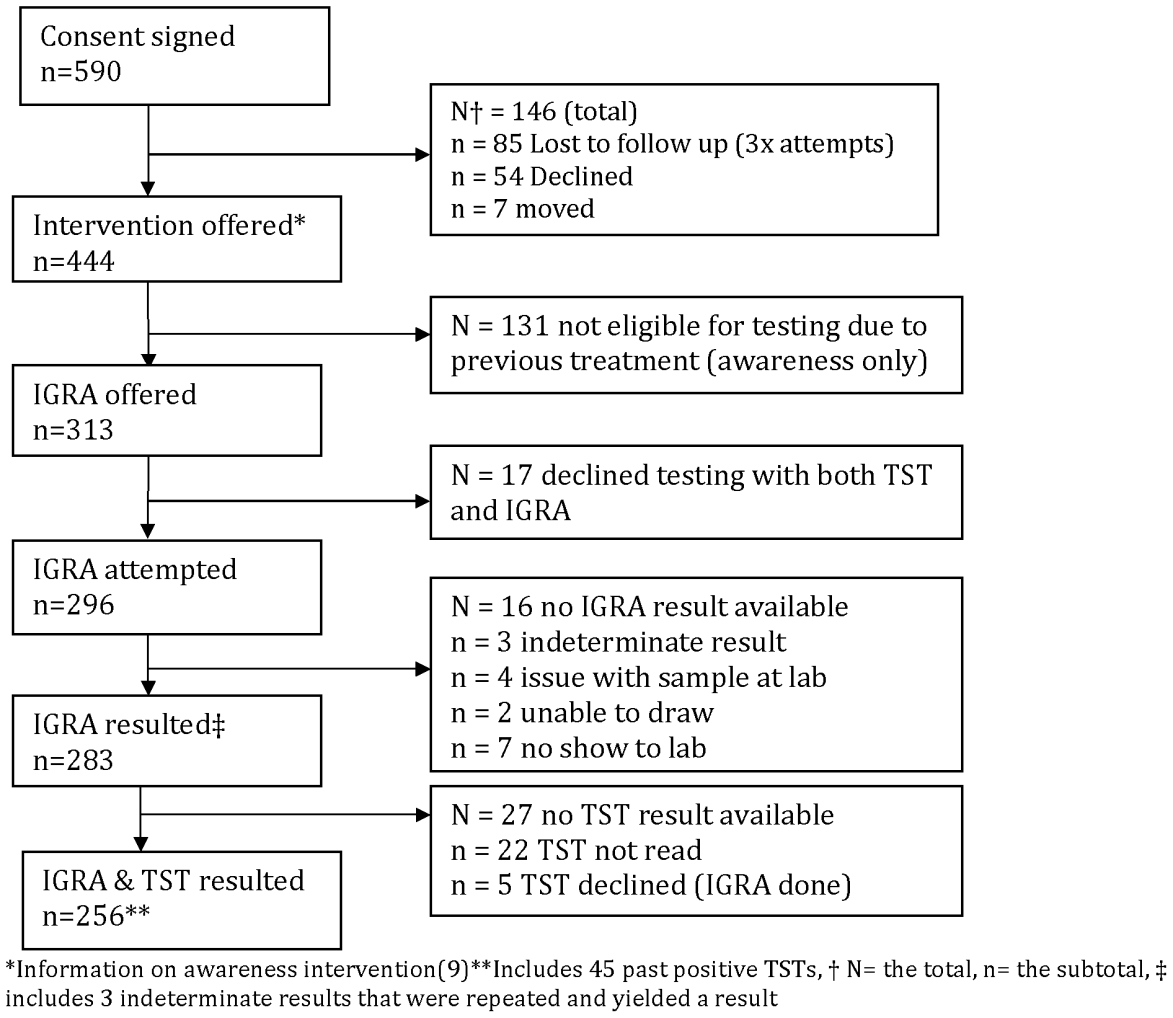
Flow chart of all participants approached.

See Table 1. As part of the overall Taima TB program [9], 590 persons signed consent to enter the study which included in home TB education followed by screening with TST and IGRA (Figure 1). Results were obtained for 94.6% (280/296) of IGRAs and 91.1% (224/246) of TSTs (p value  = 0.11).

**Table 1:** Demographics of Iqaluit residents who accepted screening for LTBI in residential areas of high risk for TB*.

Characteristic	N = 296 (%)
Age	Mean 25.4 years (23.3,27.5, IQR) Median 22.7 years(8.7,39.4, IQR)
Gender	159 females (54%)
**Origin**	
Inuit	251 (86%)
Canadian born non Aboriginal	45 (14%)
**BCG status** **	
Vaccinated	206 (73%) (79.7% of those that had records were vaccinated)
Not Vaccinated	55 (18.6%)
Unknown (no records)	35 (11.8%)
**BCG timing of vaccine (n = 206)**	
Infancy	181
Post infancy	15
Repeated vaccination	10

*More detailed description of the population [9].

**BCG vaccination was recorded only if records indicated that the vaccine was given or not given.

Of the blood samples drawn for IGRA, 5.4% (16/296) did not provide a result, 3 were indeterminate results (all yielded a result upon retesting but were counted as result not obtained initially), 6 were related to laboratory issues (sample lost by laboratory (n = 2), incubation problem (n = 2), unsuccessful phlebotomy (n = 2 children)) and 7 were related to participant issues (no show at laboratory n = 7 children). Of the TSTs that were implanted, results were not obtained in 22/246 subjects (8.9%), because these subjects were lost to follow up or their TST was not read within the specified 48–72 hours. IGRA testing resulted in more available test results reaching patients (95.6% vs 90.9% p = 0.02). Time to result was a median of 8 days for IGRA versus 2 days for TST, p value  = <0.0001.

Overall, 18% (52/283), (denominator includes 3 samples that were indeterminate that were repeated and yielded a result) of IGRAs were positive and 32% (87/269, denominator includes 45 previously positive untreated TST participants) of TSTs were positive. Either the TST or IGRAs were positive in 31% (93/296) of participants. Of the 296 participants, 256 (86%) had results of both tests available for measure of test concordance. Reasons for not having both test results available: 5 subjects declined the TST, 13 subjects had no IGRA result available after re-testing and 22 subjects did not have a TST result read within 72 hours. See Table 2.

**Table 2:** TST and IGRA concordant and discordant results.

IGRA				
		Positive	Negative	Totals
**TST**	Positive	46 (18%)	**40 (15.6%)**	86
	Negative	4 (1.6%)	166 (64.8%)	170
	Totals	50	206	256

Total concordance of both tests was 82.8%. Total discordance for either scenario was 17.2% with the majority of the discordance seen in the TST+/IGRA – pattern (15.6%). Inter test reliability Kappa score agreement between TST and IGRA (Kappa = 0.57, 95% CI (0.46–0.68), p value <0.0001).

The univariable logistic regression model showed a significant association of discordance between the TST and IGRA with age and BCG. BCG given post infancy or repeated BCG was associated with discordance (Table 3). BCG given in infancy was not significantly associated in the univariable regression model but did become significant once gender, ethnicity and repeated TSTs were added to the multiple logistic regression model (Table 4). Furthermore, age and BCG given at infancy, post infancy and repeated BCGs demonstrated a step wise increase in association to discordance compared to no BCG (Table 4).

**Table 3:** Univariable logistic regression model for predictors of discordance between the TST and IGRA (TST+/IGRA− compared to TST−/IGRA−) (n = 38).

Characteristic compared	Relative Risk	95% CI	p-value
Age (1 year increase)	1.03	1.01–1.04	0.0003
Inuit vs Canadian born non Aboriginal	1.62	0.68–3.86	0.2749
Female vs Male	0.62	0.35–1.09	0.0969
BCG status yes vs No	3.39	1.08–10.62	0.0360
BCG at infancy* vs No BCG	2.76	0.87–16.02	0.0861
BCG post-infancy vs No BCG	6.96	1.87–25.97	0.0039
Repeated BCG vs No BCG	10.44	3.05–35.82	0.0001
Repeated BCG vs BCG at infancy	3.77	1.90–7.50	0.0001
Repeated BCG vs Post-infancy	1.50	0.59–3.78	0.3897
Post-Infancy BCG vs Infancy	2.52	1.10–5.76	0.0286
10 year increase in time since last BCG	1.36	1.11–1.64	0.0019
1 increase in # of TST	1.00	0.92–1.11	0.8378
≥3 TSTs vs <3 TSTs (all TSTs)	1.42	0.82–2.48	0.2118
≥3 TSTs vs<3 TSTs (done within 4 years)	0.55	0.14–2.09	0.3804

*defined as the 1^st^ year of life.

**Table 4:** Multivariable logistic regression model for predictors of discordance between the TST and IGRA (TST+/IGRA− compared to TST−/IGRA−) adjusting for gender, ethnicity and repeated TSTs (n = 38).

Characteristic	Relative Risk (95 CI, p value)
Age (1 year increase)	1.04 (1.00–1.06, p = 0.006)
BCG at infancy vs no BCG	6.43(1.72–24.85, p = 0.005)
BCG post infancy vs no BCG	8.13(2.54–26.03, p = 0.0004)
BCG repeated* vs no BCG	20.03(3.94–101.82, p = 0.0003)

*BCG repeated refers to more than one BCG vaccine given on separate occasions.

As part of the sensitivity analysis, multivariable regression analysis was run separately with a comparison of discordant pairs with both concordant negative and positive pairs and the only difference noted was that in the multivariable regression age was no longer significant however all three BCG categories remained significant.

## Discussion

In this population of predominantly young Inuit who were mostly BCG vaccinated in infancy and residing in residential areas of high risk for TB, the use of IGRA for the diagnosis of LTBI was feasible. Use of this test resulted in more available test results reaching patients compared to the TST. A relatively large percentage of subjects tested TST positive, but were IGRA negative. Multivariate analysis suggested that participants with a discordant result were much more likely to have received multiple BCG vaccinations, followed by BCG given post infancy and then to a lesser degree when BCG was given in infancy. This suggests that the TST may be falsely positive in patients with a history of previous BCG vaccine, even if given in infancy. In this situation, use of the IGRA test may increase specificity of testing for latent TB. Use of the IGRA test for the BCG vaccinated population in Canada's northern communities could potentially reduce the number of persons being offered preventative LTBI treatment.

A significant number of results were not obtained using the TST compared to the IGRA (9.8% vs 5.4% p = 0.02) despite rigorous study protocols and use of experienced public health nurses. This occurred since there is only a finite window period when the TST can be read whereas the IGRA does not have this limitation. Under study conditions this may have resulted in missed treatment opportunities in the group that did not have the TST read. The protocol that was developed circumvented the lack of laboratory staff to run the test in Iqaluit by sending the samples to the reference laboratory in Ottawa after their blood had been incubated and centrifuged in Iqaluit. The IGRA results took longer to arrive back to the patient compared to the TST (mean 8 days versus 2 days, p value  = <0.0001). This occurred as a result of the IGRA test being run once a week in the reference laboratory. Flight schedules also lengthened the time from blood draw to results. The IGRA result could potentially be obtained faster if the Iqaluit laboratory had the staff to run the test on-site. IGRA testing did not provide a result in 5.4% (1% indeterminate and 4.4% did not yield results) of people tested in the present study which is comparable to other studies [6], [15]. IGRA indeterminate results were rare and retesting resulted in results obtained. Phlebotomy challenges with young children did not impede obtaining IGRA results in most cases. Compared to a previous feasibility study done in a public health clinic in Baltimore [6] the failure rate was 3.25% (0.25% indeterminate and 3% did not yield results). In another study [15] done in six community clinics serving homeless, immigrant and injection drug users in San Francisco the failure rate was 7% (2% indeterminate and 5% did not yield results).

In a comparable Inuit population in Greenland [16], IGRA was introduced for screening Inuit children for LTBI and a much lower discordance rate was observed (2.4% Greenland study versus 13.4% Iqaluit study (adjusted to a ≥12 mm positive TST used in Greenland study). One reason for the increased discordance seen in the present study may be related to the differences in BCG vaccination rates. In the present study, 79.7% of those with a full immunization record, were vaccinated compared to 34.8% in the Greenland study [16]. Kappa agreement with BCG at infancy was calculated at 0.68 in the Greenlandic study [16] compared to 0.58 in the current study. Low BCG vaccination rates in the tested population increase both concordance and agreement between the two tests [11].

In order to understand the BCG effect with greater clarity we then studied the timing of the vaccination (infancy versus post infancy), and we studied the effect of multiple vaccinations given to the same individual and its effect on discordance between the two tests (Table 4). A gradation of the effect of timing of BCG is demonstrated in the present study. After adjusting for age, ethnicity, gender and repeated TSTs, multiple BCG vaccinations showed the strongest association with a discordant result, followed by BCG given post infancy and then to a lesser degree BCG given in infancy. In the 1960s, multiple BCG vaccinations were common and given 3–4 times during the first 5–10 years of life in Nunavut (formerly part of the North West Territories) [17]. In the present study, BCG vaccination was given twice in 7 participants and three times in 3 participants, all of whom were born between 1960–70.

Discordance between the two tests remained significant even if BCG was given in infancy (RR 6.43, 95% CI, 1.72–24.85, p = 0.005) (Table 4). A finding that was also described in the Greenland study, where BCG was also given in infancy (RR 2.66, 95% 1.53–4.68, p<0.0001) [16]. However, a meta analysis on the effect of BCG on TST [18] suggested that if BCG is given in infancy it should not cause false positive TSTs when the TST is done>10 years from BCG vaccination. Our findings suggest that if BCG is given in infancy the association with discordance remains. No significant difference in discordance was noted between participants who were <10 years compared to those that were >10 years. The finding may be explained by the fact that we used the medical records to determine BCG vaccination however, most of the studies included in the meta analysis used BCG scar as the method of verification of BCG vaccination instead of medical records that indicated BCG was actually given. In 20–50% of BCG vaccine recipients a scar will fail to develop [13], [14].

A recent study [19] was done in a cohort of almost 4000 First Nations school children (ages 5–13 years), who were at low risk of TB infection, of which half were BCG vaccinated *in infancy* and half were not. Vaccinated children in that study were 28.5 times more likely than non-vaccinated children to be TST positive (5.7% vs 0.2% p <0.001) suggesting that BCG given in infancy had an effect on TST in this population. Only 5/65 BCG vaccinated children who were TST positive were IGRA positive in that study suggesting that the TST may be unreliable in this population.

IGRA studies have been limited by the lack of a gold standard test to confirm latent TB infection. A proxy for the gold standard test for LTBI diagnosis is to determine the IGRA's predictive value for incident active TB. In a systematic review and meta-analysis, including 15 studies and a cohort size of 26,680 participants who were at high risk for TB and followed for a median duration of 3 years for incident active TB disease [20], neither the TST nor the IGRA had a high accuracy for the prediction of active TB. In fact most IGRA positive or TST positive participants did not progress to active TB disease, underscoring the relative lack of predictive power of both tests. However, IGRAs were shown to reduce the number of people considered for preventative treatment, especially in BCG vaccinated populations. In our study, use of the IGRA test could have potentially reduced the number of people who would have been offered preventative treatment by 15% (TST+/IGRA−) according to the current standard of care using TST only. Given the risk of side effects which in a young population is low but nonetheless not trivial, the cost of treatment in this population, and the significant lack of human resources in health care in Nunavut, reducing the number of people requiring 9 months of directly observed therapy may be of programmatic benefit. The lack of human resources is accentuated further in the rest of communities in Nunavut which are all smaller than Iqaluit. Furthermore the high turnover of staff results in less experienced staff having to implant and interpret TSTs, a problem that was not encountered with IGRA use. Another reason to use this test in Nunavut is the amount of time the TST consumes from the nurses. Currently public health nurses do the TST implantation on the first visit and then interpret the results on the second visit. Many people return to have their TSTs read at the health center but often nurses are left trying to find the individual to interpret the results.

This study has several potential limitations including the issues of generalizability of our study results to the entire northern Canadian population. This study employed a door to door screening and testing campaign in residential areas of high risk for TB within one of the largest urban community in Canada's north. The LTBI cases diagnosed in this study represent extra cases of LTBI found outside of the contact tracing around active TB cases which is traditionally conducted by the public health program in Iqaluit. The prevalence of latent TB infection in our high-risk group would be expected to be greater than the overall prevalence of latent TB in the general Nunavut population. There may be variables that are yet undetermined which affected the discordance of these two tests. Although non tuberculous mycobacteria (NTM) can cause false positive TSTs and could have contributed to test discordance [11], this information was not collected and little is known about the prevalence of NTM organisms in this population. Our study was not powered to show a difference in the number of people starting treatment according to which test was done since most of the participants got both tests. We did not collect information on exposure time with an active TB case, tuberculin skin test type or BCG vaccine subtype. Conversion (negative to positive) and reversions (positive to negative) in serial IGRA testing have been noted and have caused significant concern for the utility of IGRA in serial testing of health care workers [21], [22] however recent evidence in a study of adolescents in a high TB incidence region, suggests that conversion of IGRA from negative to positive is associated with an eight fold increased incidence of active TB over a two year period following conversion compared to those that did not demonstrate IGRA conversion [23]. None of the reversions (positive to negative) developed active TB during the study period [23]. A formal cost effectiveness study will be done to analyze if the decreased number of people requiring treatment in this population outweighs the increased cost of the IGRA compared to the TST.

In conclusion, our study suggests that IGRA testing is feasible. IGRA results are returned in 95% of subjects tested in a medically underserved remote arctic Aboriginal population in Canada's north. In our population there was a relatively high rate of test discordance between the IGRA test and the TST, likely explained by the high prevalence of previous BCG vaccination in our subjects. IGRA testing in Canada's northern population may potentially offer a more specific method to test for latent TB and could result in fewer people having to initiate latent TB treatment with INH in a region with limited public health human resources.
